# Supplementary figures and images for: Aspergillus fumigatus transcription factor ZfpA regulates hyphal development and alters susceptibility to antifungals and neutrophil killing during infection
Source: bioRxiv. 2023 Jan 26:2023.01.25.525624. Preprint. [Version 1] doi: 10.1101/2023.01.25.525624 (PMC9901008; doi:10.1101/2023.01.25.525624)

675 S1 Fig

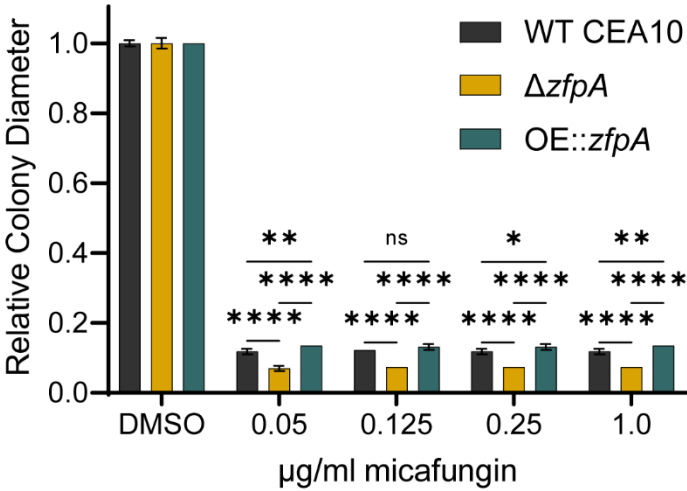

676

677 S2 Fig

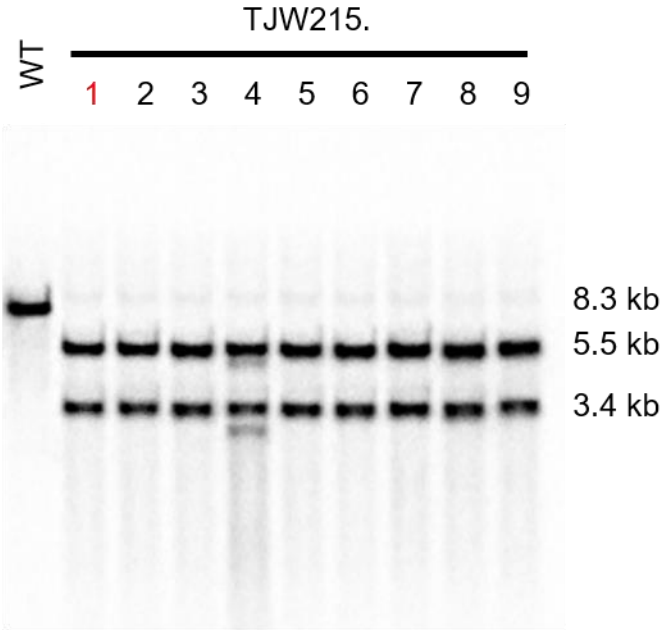

678

679 S3 Fig

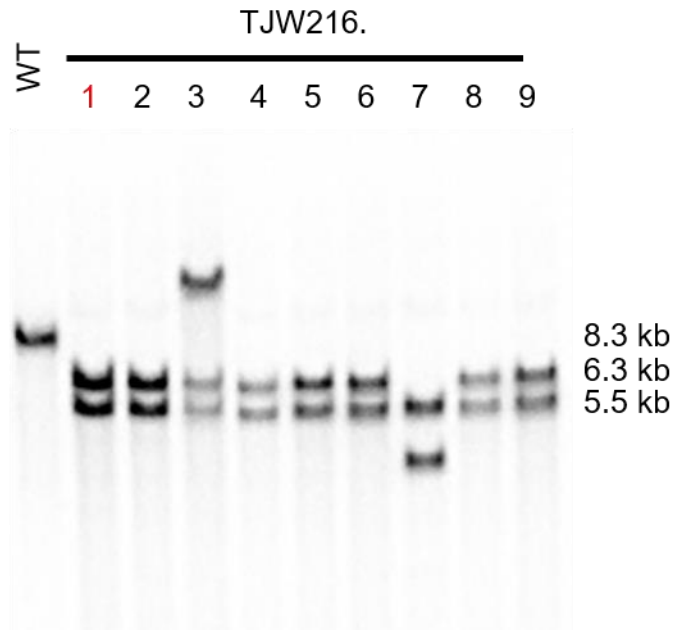

680

681

Supplement: 1 — S1 Fig. ZfpA mediates micafungin tolerance. Susceptibility of WT CEA10, ΔzfpA, and OE::zfpA to 0.05, 0.125, 0.25, and 1 μg/mL micafungin. 104 spores were point-inoculated on solid GMM with micafungin or DMSO. Bars represent mean±s.d. of colony diameter at 4 days post inoculation of 4 plates per condition. p values calculated by ANOVA with Tukey’s multiple comparisons. *p<0.05, **p<0.01, ****p<0.0001. S2 Fig. Southern confirmation of ΔzfpA mutants. Genomic DNA was digested by PciI. Wild type (8.3 kb), and ΔzfpA (5.5 and 3.4 kb). TJW215.1 was chosen for the subsequent experiments. S3 Fig. Southern confirmation of OE::zfpA mutants. Genomic DNA was digested by PciI. Wildtype (8.3 kb), and OE::zfpA (6.3 and 5.5 kb). TJW216.1 was chosen for the subsequent experiments. Movie S1: Interactions between neutrophils and wild-type CEA10 germlings. Representative movie of neutrophils engaging with two WT CEA10 germlings. One germling loses cytoplasmic RFP signal and is killed while the other escapes surrounding neutrophils. Images were acquired every 3 min for 12 h. Left panel: brightfield. Right panel: A. fumigatus cytoplasmic RFP. Scale bar = 20 μm. 10 frames/s. Movie S2: Interactions between neutrophils and ΔzfpA germling. Representative movie of neutrophils engaging with ΔzfpA germling. The germling does not escape surrounding neutrophils and loses cytoplasmic RFP signal within 30 min of co-incubation. Images were acquired every 3 min for 12 h. Left panel: brightfield. Right panel: A. fumigatus cytoplasmic RFP. Scale bar = 20 μm. 10 frames/s. Movie S3: Interactions between neutrophils and OE::zfpA germlings. Representative movie of neutrophils engaging with two OE::zfpA germlings. One germling does not escape surrounding neutrophils and loses cytoplasmic RFP signal after 225 min of co-incubation while the other escapes. Images were acquired every 3 min for 12 h. Left panel: brightfield. Right panel: A. fumigatus cytoplasmic RFP. Scale bar = 20 μm. 10 frames/s. Movie S4: Neutr [file NIHPP2023.01.25.525624v1-supplement-1.pdf]
